# Water Footprint Assessment of Selected Polymers, Polymer Blends, Composites, and Biocomposites for Industrial Application

**DOI:** 10.3390/polym11111791

**Published:** 2019-11-01

**Authors:** Jerzy Korol, Aleksander Hejna, Dorota Burchart-Korol, Błażej Chmielnicki, Klaudiusz Wypiór

**Affiliations:** 1 Department of Material Engineering, Central Mining Institute, Pl. Gwarków 1, 40-166 Katowice, Poland; kwypior@gig.eu; 2 Department of Polymer Technology, Gdansk University of Technology, Narutowicza 11/12, 80-233 Gdansk, Poland; 3 Faculty of Transport and Aviation Engineering, Silesian University of Technology, Krasińskiego 8, 40-019 Katowice, Poland; dorota.burchart-korol@polsl.pl; 4 Paint & Plastics Department in Gliwice, Institute for Engineering of Polymer Materials and Dyes, 50 A Chorzowska Street, 44-100 Gliwice, Poland; b.chmielnicki@impib.pl

**Keywords:** water footprint, environmental impact assessment, biocomposites, natural fibers

## Abstract

This paper presents a water footprint assessment of polymers, polymer blends, composites, and biocomposites based on a standardized EUR-pallet case study. The water footprint analysis is based on life cycle assessment (LCA). The study investigates six variants of EUR-pallet production depending on the materials used. The system boundary included the production of each material and the injection molding to obtain a standardized EUR-pallet of complex properties. This paper shows the results of a water footprint of six composition variants of analyzed EUR-pallet, produced from biocomposites and composites based on polypropylene, poly(lactic acid), cotton fibers, jute fibers, kenaf fibers, and glass fibers. Additionally, a water footprint of applied raw materials was evaluated. The highest water footprint was observed for cotton fibers as a reinforcement of the analyzed biocomposites and composites. The water footprint of cotton fibers is caused by the irrigation of cotton crops. The results demonstrate that the standard EUR-pallet produced from polypropylene with glass fibers as reinforcement can contribute to the lowest water footprint.

## 1. Introduction

Global production and consumption of plastics have been continuously increasing over the last decades. Plastics are successfully replacing other materials, e.g., metals, wood, paper, ceramics, and glass. This phenomenon is associated with the specific properties of plastics, such as low density, corrosion resistance, and simplicity of processing, which requires a noticeably lower energy input in comparison to metals or ceramics. Various branches of industry, especially those in which advanced technologies are applied, e.g., cosmonautics, medicine, and informatics, are developing very rapidly thanks to novel polymeric materials. Plastics are commonly applied in innovative technological solutions and novel design, such that they have become an integral part of the surrounding world. Nevertheless, with increasing plastics production and multiplicity of their common applications, the amount of generated waste is also significantly growing, which is very aggravating for the environment and is becoming a noticeable problem at global scale. A natural consequence of this situation is increased interest in biodegradable materials, which, in contrast to conventional plastics produced from petrochemical-based raw materials, can be decomposed with noticeably less harmful effects on the natural environment. These materials include mainly biopolymers, starch and its derivatives, biopolyesters, which can be produced via fermentation of polysaccharides, e.g., polyhydroxybutyrate (PHB) or poly(lactic acid) (PLA). Except polymers produced from natural resources (mainly plant-based), there are also known synthetic, biodegradable polymers produced from petroleum, such as polycaprolactone (PCL), poly(vinyl acetate) (PVA), or poly(ethylene glycol) (PEG). However, currently costs of their production are considerably higher compared to conventional synthetic polymers, so their manufacturing on a global scale is still limited. Other types of polymeric materials, which are very interesting from an environmental point of view, are polymer blends and composites, which involve simultaneous use of synthetic polymers and biodegradable materials—polymers or natural fillers.

In the scientific literature, especially regarding material engineering, the topic of biocomposites is discussed very often. There are a lot of reports related to their manufacturing, performance, potential uses, and possibilities of biodegradation. Decomposition of biodegradable components in such materials results in the loss of their integrity, which often facilitates their postconsumer treatment. These materials, after their use by consumers, tend to biodegrade or disperse, depending on their biodegradable phase. However, bio-based plastics and composites, especially thermoplastics, may be considered a threat and negatively affect the recycling process. In conventional recycling lines, applied in a lot of small and medium companies specialized in plastics’ recycling, it is very hard to precisely identify and separate bio-based plastics or to manufacture homogenous polymer blends with conventional petrochemical plastics because of their low miscibility. Only the addition of a proper compatibilizer enables production of a homogenous structure of blends containing hydrophilic and hydrophobic plastics [1]. Such a phenomenon was clearly observed in the market, where traditional polyethylene terephthalate (PET) bottles were replaced by bio-based PLA ones. A stream of PET bottles was contaminated with incompatible PLA [2]. It can have a negative influence on the circular economy. On the other hand, bioplastics enable reduction of demand for fossil fuels and limit the negative environmental impact of their extraction and processing. Moreover, the majority of bio-based plastics tend to degrade in the natural environment significantly faster than conventional plastics, which is obviously very beneficial for the environment, e.g., by reducing the phenomenon of “waste islands” in oceans [3,4,5]. Hence, their popularity is currently growing, so in the near future they may become a real issue in terms of their recycling and utilization.

Despite a multiplicity of works associated with the structure and performance of bio-based plastics, blends, and composites, in the case of their environmental impact and footprint, the number of reports is noticeably lower. There are some works discussing the evaluation of biocomposites’ environmental impact on the basis of life cycle analysis [6,7] or eco-effectivity analysis [8]. In the work of Vilaplana et al. [9] there are presented the most important environmental aspects associated with engineering of sustainable biocomposites. Martinez et al. [10] and Vidal et al. [11] performed the life cycle assessment of biocomposites based on high-density polyethylene (HDPE) and polypropylene (PP) filled with rice husk and waste cotton fibers. Yates and Barlow [12] presented the life cycle analysis of production processes of various biopolymers, e.g., PLA, polyhydroxyalkanoates (PHA) or thermoplastic starch (TPS). Moreover, they presented comparative analysis of the environmental impact of production of biopolymers and petroleum-based polymers, such as PET, polystyrene (PS), polyethylene (PE), or PP. The authors stated that, despite the numerous reports, it is hard to clearly tell which one of the analyzed materials is characterized with the lowest environmental impact. This was due to the fact that, despite the biomaterials showing a lower demand for nonrenewable energy and resources and a lower greenhouse effect compared to petroleum-based plastics, they were characterized with a noticeably higher impact related to land use or eutrophication, just as in the case of starch-based plastics investigated by Broeren et al. [13]. However, observed trends indicate that environmental footprints of biopolymers and biocomposites are becoming more and more beneficial over time [14]. Nevertheless, the analysis presented by Yates and Barlow [12] was related only to the production process of analyzed materials without their further use. Because of the widely anticipated and systematic increase in global demand for polymeric materials, currently it is very important to develop effective and safe methods of their utilization [15]. Manufacturing of biodegradable plastics intended for disposable packaging materials is one possibility to prevent environment pollution and to reduce the amount of plastic waste, whose degradation is very slow. Therefore, Alvarez-Chavez et al. [6] analyzed the health and environmental impact of bio-based plastics and developed a “bioplastic spectrum”, which should facilitate the selection of bioplastic. It was found that it is not possible to unambiguously indicate what material is the most environmentally friendly and if it fully meets the criteria of sustainable development. Bio-based raw materials and products derived from them are preferred because of their lower impact towards human health and the environment during their use. Among analyzed materials, the most beneficial in terms of the environmental impact were TPS and PLA.

Nevertheless, a noticeable issue of bio-based plastics in terms of environmental impact is the source of raw materials. Cultivation of crops for industrial uses often burdens the environment, exhausts its resources, and causes climate changes. It is associated with land management, application of fertilizers and chemicals, such as pesticides or herbicides, as well as irrigation of crops, which in the whole life cycle of biocomposites, determines their environmental impact. Depending on the considered aspects, comparison of bioplastics and conventional petrochemical-based ones in terms of their environmental impact may vary somehow. In some cases, bio-based polymers may show a bigger influence on the environment than petroleum-based plastics [16]. Therefore, it is very important that in the case of bioplastics, the assessment of their environmental impact is not limited only to the use of raw materials, nonrenewable energy, and emission of greenhouse gases, as in the majority of works [17]. Other important issues, such as the evaluation of water use required for production and processing of bio-based plastics, should be also addressed and analyzed, because fresh water has become one of the rarest and most exploited natural resources [18]. For this purpose, various indicators are used, from which one of the most popular and well described in the literature is a water footprint.

Water footprint is a measure of drinking water used by humans. A product’s water footprint is then a total volume of drinking water used to manufacture a product throughout the whole production process [19]. It is divided into the following components: blue, green, and grey. Blue measures the volume of used surface water and groundwater. Green water footprint is related to the use of rainwater, unless it is drained into sewers. The increase of use of rainwater reduces the blue water footprint. When rainwater is drained into sewers, it can be contaminated with sludge, generating grey water footprint. Additionally, rainwater runoff from farmlands, which contains pesticides or fertilizers, increases the grey water footprint, which is defined as the volume of fresh water required to assimilate pollutants to meet specific water quality standards. It considers point-source pollution discharged to a freshwater resource.

Water footprint measures not only the direct use of fresh water by producer or consumer, but also indirect uses during the whole life cycle of the product or process [20]. It can be perceived as a universal indicator of fresh water resources, which determines the use of fresh water from various sources and amount of water contaminated by the type of pollutant. All components of a water footprint are specified from the geographic and time point of view. Use of water is associated with the loss of surface water and groundwater in a particular area. Losses are generated by evaporation of water, outflow to other areas or reservoirs, or by manufacturing of the product. As indicator water use, water footprint differs from the classic measure of water intake. Water footprint offers a better and wider perspective on what is actually the role of consumers and manufacturers in the water use. Calculations of water footprint provide the information about how water is used in various human activities, as well as various industrial processes and by various products.

Generally, demand for fresh water increases rapidly with climate changes, increase of population, constant economic development, and expansion of crops used for production of biofuels and bioplastics, which concerns both governmental and nongovernmental organizations. As a result, about a decade ago, many companies from various sectors started to measure and report a negative environmental impact related to water used for production. Earlier, water footprint was determined mainly for cotton [21], coffee and tea [22], meat [23], tomatoes [24], and other food products [18]. Reports are published more and more often in media, and the term water footprint has become almost as popular as carbon footprint.

However, literature reports related to the calculation and evaluation of water footprints are associated mainly with the agricultural industry, since 69% of global water withdrawal is used in agricultural production [25]. Nevertheless, agricultural products are often used as intermediate inputs in other industries, such as food, textile, chemical, or, lately more and more often, in the plastic industry, which is caused by growing interest in bio-based polymers, as well as polymer blends and composites. Therefore, incorporation of raw materials of agricultural origin into these sectors significantly affects the water footprint of resulting products. According to multiple literature reports [26,27], unitary industrial use of water related to metals, plastics, or ceramics and water footprint of these materials is noticeably lower compared to bio-based products, which are more and more often used as their replacement in various applications. As mentioned before, except noticeable benefits, incorporation of bio-based raw materials into the plastics industry could in some way unfavorably affect the environmental impact of plastics.

It is also very important to remember that despite the relatively low popularity of bioplastics, their blends, and biocomposites nowadays, their application will grow and, in the future, will have a large impact on the natural environment. Therefore, it is important to determine their environmental impacts, including water footprint, now, in the relatively early stage of their development in order to protect the environment in the future. This was confirmed by Ercin and Hoekstra [28], who presented four different scenarios for global water footprint for 2050 depending on globalization, economic development, environmental sustainability, and regional self-sufficiency. Their analysis showed that long-term changes in water footprint are very sensitive to multiple factors, especially consumption patterns, economic growth, and technology development in various sectors. For this reason, it is important to analyze the water footprint of various industrial products, in order to engineer future technologies avoiding unnecessary water usage.

Having in mind the above mentioned trends and factors related to the environmental impact of plastics, the main goal of the presented study was to calculate and evaluate the water footprint of a commonly applied material—polypropylene, as well as its blends with bio-based polymers (poly(lactic acid) and thermoplastic starch) and composites with natural fibers (cotton, jute, and kenaf fibers), whose popularity is increasing over the last decades. Analysis was performed for widely applied in various branches of industry standardized plastic pallet. Presented research shows interdisciplinary character, combining aspects of environmental and materials engineering. On the basis of performed evaluations of environmental impact of biopolymers and biocomposites, considering various aspects, it aims to answer the following question—Can the application of bioplastics and biocomposites obtained from renewable resources as engineering materials reduce the negative environmental impact compared to conventional petroleum-based plastics showing similar processing and performance?

## 2. Experimental

### 2.1. Aims and Scope of Analysis

The main target of the presented work was analysis and evaluation of the water footprint of selected polymer blends, as well as composites containing glass and natural fibers.

**Figure 1:**
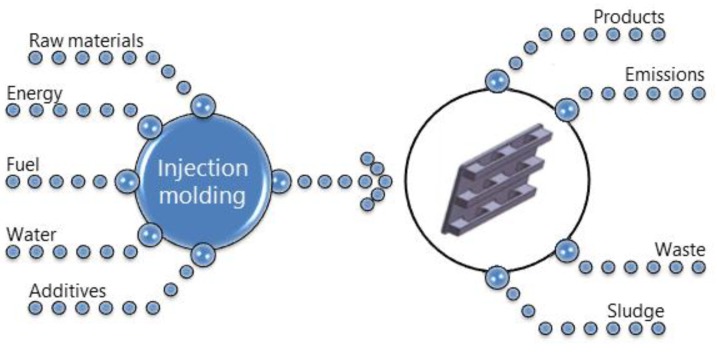
Scheme showing scope of performed analysis.

Analysis was performed according to cradle-to-gate assessment, and boundaries were set from raw materials stage to final product obtained by injection molding technology, which is schematically presented in Figure 1. All input and output streams were included in analysis. Recycling of post-consumer pallets was not included.

**Figure 2:**
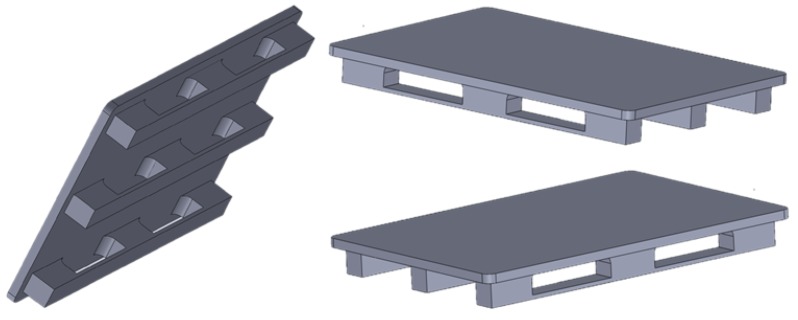
Standard EUR-pallet.

For the purpose of analysis, one standard EUR-pallet was selected as the functional unit (FU), according to the standards of the European Pallet Association, as shown in Figure 2. The mass of the analyzed pallet is 15 kg.

Plastic pallets are commonly and more and more often applied in various branches of industry. This effect is related mainly to their good performance properties, ease of cleaning, and noticeably longer estimated life compared to wood pallets [29]. A multiplicity of commonly used plastics and plastic-based composites, as well as constant development of technologies of their manufacturing and processing, enable adjustment of proper material for the desired application of the pallet. The most popular plastic used for production of pallets is polypropylene (PP), mainly because of its good mechanical parameters and low price. Moreover, various modifications of PP can be relatively easily applied, e.g., by incorporation of various fillers, such as mineral particles or glass fibers [30]. PP is the most commonly used thermoplastic polymer in Europe as a matrix for composites. Moreover, due to its hydrophobic character, it is a very good barrier for humidity, which can affect the structure and performance of composites containing natural fibers or blends with biodegradable plastics [31,32]. Therefore, PP was chosen as the main polymer matrix in presented research.

**Figure 3:**
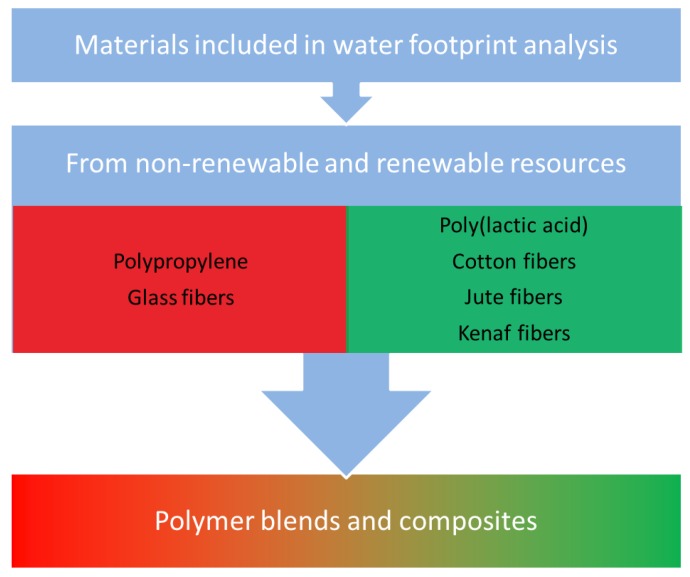
Materials included in water footprint analysis.

As mentioned above, PP can be easily and effectively filled with various fillers, often fibrous ones, which often offer excellent enhancement of mechanical performance, such as widely applied glass fibers. An alternative for the conventional fillers are natural fibers. These resources are mostly plant-based, and therefore often are renewed every year, which results in their relatively low price and good availability [33,34,35]. Application of bio-based raw materials in plastics technology is one of the ways to reduce the use of nonrenewable resources, e.g., fossil fuels. According to life cycle assessment, incorporation of natural fibers often allows the reduction of the environmental impact of plastic-based products [8,36]. Moreover, such biocomposites can also show very good mechanical properties, which can be tailored by the proper selection of filler and its parameters. Use of lightweight natural fibers allows reduction of the materials’ density, which is very beneficial, e.g., in building or automotive applications [37]. Another alternative for petrochemical-based polymers, except the application of natural fillers, is the preparation of polymer blends, including biodegradable polymers, such as poly(lactic acid) (PLA), polycaprolactone, or thermoplastic starch, which also reduce the use of nonrenewable raw materials. A big advantage of biocomposites and polymer blends based on polypropylene, polyethylene, or other “conventional” polymers is the ability to obtain materials resistant to weather conditions and moisture. Therefore, enhancement of environmental factors may be achieved with simultaneous maintenance or improvement of mechanical performance. Over the last years, a noticeable increase in interest in biocomposites and polymer blends containing biodegradable polymers may be observed [38,39]. For the purpose of the presented analysis, the following materials were selected as fillers for PP-based composites: cotton fibers (CF), jute fibers (JF) and kenaf fibers (KF), as well as glass fibers (GF), which are one of the oldest reinforcement used in manufacturing of polymer composites [40]. Nowadays, glass fibers are often replaced by natural fibers in the production of composites, e.g., in the automotive industry [41,42]. Except for composite variants, use of PLA as a component for PP-based polymer blends was investigated, since blends of these two materials often show comparable or even superior properties to neat PP with simultaneous enhancement of environmentally-friendly character [43]. Additionally, PLA is among the most commonly used bio-based plastics in Europe [44]. A scheme of variants included in the analysis is presented in Figure 3.

It is important to remember that when a certain content of biofiller or biodegradable polymer in composite or blend is achieved, the material can become partially biodegradable. However, in case of analyzed pallets, biodegradability is undesirable. Therefore, based on literature reports and results presented in our previous works, six variants of composition of analyzed materials were developed [45,46]. The content of natural fibers in analyzed composites was fixed at 30 wt %, which allows one to maintain or enhance the mechanical properties of material, compared to neat PP. According to literature data, the tensile strength of unmodified PP is in the range 25–33 MPa, and Young’s modulus is in the range 1000–1400 MPa. Addition of 30 wt % of natural fibers can enhance these parameters to the levels of 40–45 MPa and 3000–3500 MPa, respectively, depending on the type of filler, as well as shape and size of fibers [47]. Except for the application of natural fibers, variant when PP was blended with 30 wt % of PLA, which shows superior mechanical properties, was analyzed. For comparison with environmentally-friendly material solutions, conventional variant with 10 wt % addition of commonly applied glass fibers, which results in similar mechanical performance of composite, was also analyzed.

Table 1 shows the analyzed formulations of raw materials for the manufacturing of EUR-pallets.

### 2.2. Methodology of Water Footprint Calculation

Evaluation of water footprint is one of the newest methods for determination of environmental impact of product or technology. Basics for the methodology were developed by Hoekstra in 2003 [48], and since then coworkers continue its development [49,50]. As mentioned above, water footprint includes not only direct use of water by producer and consumer, but also indirect uses, which are associated with operations such as irrigation, cooling, processing of raw materials, waste management, and contamination.

In the presented study, evaluation of the water footprint for an EUR-pallet was performed according to the methodology proposed by Hoekstra et al. [51], suggesting that complete evaluation of water footprint includes four key steps:

• establishing of goals and scope of analysis,
• accounting of water footprint,
• assessment of water footprint sustainability,
• formulation of water footprint response.

Analysis of water footprint is being undertaken for a variety of reasons. It allows determination of the dependences between economy and local water supplies. Governments may obtain information regarding the use and requirement for water from external supplies. Companies may determine their impact on water supplies and limit water utilization, becoming more environmentally-friendly. As mentioned above, in the presented study, the main goal was to evaluate the water footprint of selected polymer blends and composites, which may in the future replace conventionally applied, fully petrochemical-based polymeric materials.

According to European Commission Recommendation 2013/179/EU on the use of common methods to measure and communicate the life cycle environmental performance of products and organizations, in order to obtain consistent, reliable, and repeatable environmental footprints of products or organizations, a set of universal analytical rules used for footprint assessments should be applied [52]. These rules are aimed at providing superior guidelines for analysis of environmental impacts. They should be applied during each step of analysis, starting from formulation of goals and determination of its scope, through gathering of data, evaluation of environmental impact, and ending at reporting and verification of obtained results.

For the proper evaluation of environmental footprints, except application of recommended individual methodology for particular footprints, the following rules should also be taken into account:

- Relevance—all applied methods and data gathered for quantitative determination of environmental footprints of product should be relevant to analysis.
- Completeness—during quantitative determination of footprints, all material and energy flows, as well as other aspects, which are necessary for the compatibility with boundaries of analyzed system and requirements for the analysis and applied methods, should be considered.
- Integrity—during all steps of environmental footprint assessment of the product, compatibility with proper methodology should be maintained in order to increase integrity of analysis and comparability with similar works.
- Accuracy—all uncertainties of the system and its modelling, as well as reports related to the analysis should be limited.
- Clarity—information related to the environmental footprint of the product should be revealed in the manner that provides the recipients basis for decision-making and enables interested parties’ evaluation of their reliability and credibility.

### 2.3. Input Data

Data required for the determination of water footprint of polymer blends and composites were collected from scientific publications, reports, and databases, including Ecoinvent database v 3.1.

For the purpose of analysis of PP water footprint, the following assumptions were made:

- polymerization of propylene is performed with 95% yield,
- 75% of the production is based on suspension polymerization,
- 25% of the production is based on gas phase polymerization,
- for both types of polymerization, 4 MJ of electric energy per kg of PP and 4 MJ of thermal energy is required.

Analysis included obtaining and processing of raw materials, as well as transport and utilization of generated waste [53].

Determination of PLA granulate production water footprint was based on data obtained from the biggest producer of PLA—NatureWorks company. Data included production of raw materials, including maize and chemical modifiers and additives, transport, use of electricity during whole life cycle, and waste management [54].

Evaluation of glass fibers water footprint was based on averaged data collected from leading European glass producers. Data include analysis of 26 production lines in 12 European countries. Analysis included obtaining and processing of raw materials, including glass from recycling, transport, electricity usage, and waste management [55].

For the analysis of cotton fibers water footprint, data gathered from National Residential Efficiency Measures Database were used [56]. NREL is the organization operating in the U.S., and it is the only federal laboratory performing analysis related to development, commercialization, and implementation of eco-saving technologies, often based on renewable energy and resources. One of the areas of NREL’s activity is gathering of data required for evaluation of environmental footprints of products and technologies applied in the U.S. For the purpose of the presented study, data from NREL were complemented with information from scientific publications and technical reports related to cotton production [57].

Evaluation of jute and kenaf fibers water footprint was based on data presented by experts from Natural Institute of Research on Jute and Allied Fibre Technology in India [58]. Data concern the whole life cycle of fibers, including cultivation of crops, fertilization, application of pesticides, irrigation, harvesting, transportation, processing, and fiber production. Moreover, data were complemented with other statistics, information from scientific publications, and technical reports related to jute production [54].

## 3. Results and Discussion

### 3.1. Water Footprint of EUR-Pallet

**Figure 4:**
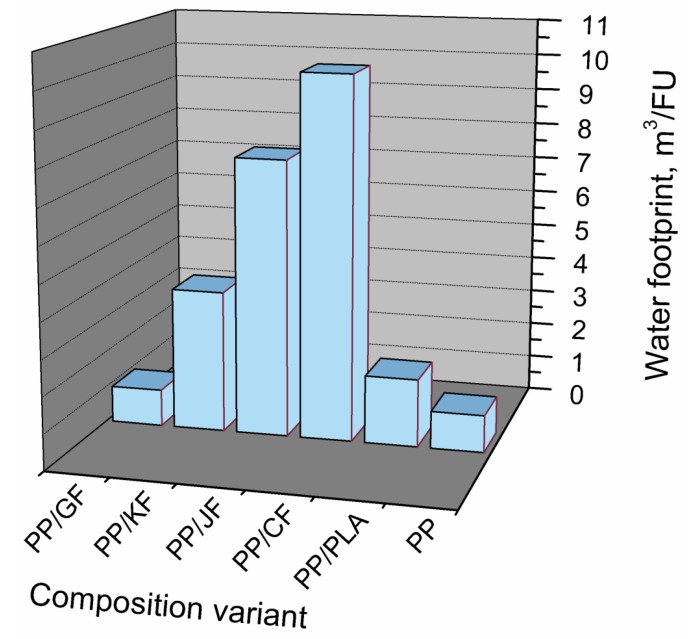
Water footprint for different composition variants of EUR-pallet.

As mentioned above, water footprint calculations are made in order to evaluate the amount of water required for manufacturing of various products. Their results for various material compositions of EUR-pallet can be seen in Figure 4. For comparison, water footprint of a pallet prepared solely from PP is also presented.

It can be clearly seen that the values of water footprint for various variants of production differ a lot. The highest values of water footprint are observed for composites filled with natural fibers—3.94, 7.73, and 10.11 m^3^/FU for kenaf, jute, and cotton fibers, respectively. Such high values are directly associated with high water demand during crop cultivation. Additionally, fertilizers and pesticides commonly used in agriculture generate high water footprints. Generally, agriculture is responsible for around 85% of total use of surface and ground water [59]. Therefore, in terms of water footprint, natural fibers can be considered a less effective modifier for PP matrix than glass fibers, whose incorporation resulted in a slight decrease of pallets’ water footprint from 1.04 to 1.02 m^3^/FU.

Basing on performed water footprint analysis, a blend with PLA is by far more environmentally-friendly than composites filled with natural fibers. Its water footprint value equals 1.89 m^3^/kg, which is several times less than for analyzed composites.

**Figure 5:**
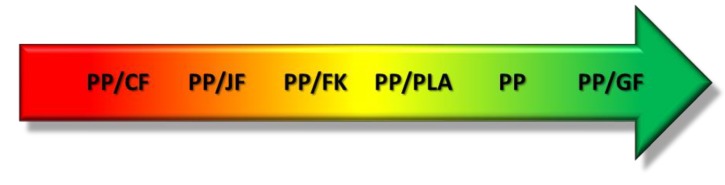
Schematic diagram of recommended material variants for EUR-pallet in terms of water footprint.

A diagram of recommended materials for the analyzed EUR-pallet was prepared basing on the results of conducted analysis, which is shown in Figure 5. Such presentation of the obtained results facilitates their clear and unequivocal understanding.

### 3.2. Water Footprint of Applied Raw Materials

The presented values of water footprint for investigated material variants of EUR-pallet may seem a bit surprising, since biocomposites, considered as an environmentally-friendly alternative for conventional plastics, are rather nonrecommended on the basis of the presented results. For clarification and better understanding of the origin of water footprint values, below are presented detailed analyses of water footprints for all analyzed raw materials.

#### 3.2.1. Polypropylene

**Figure 6:**
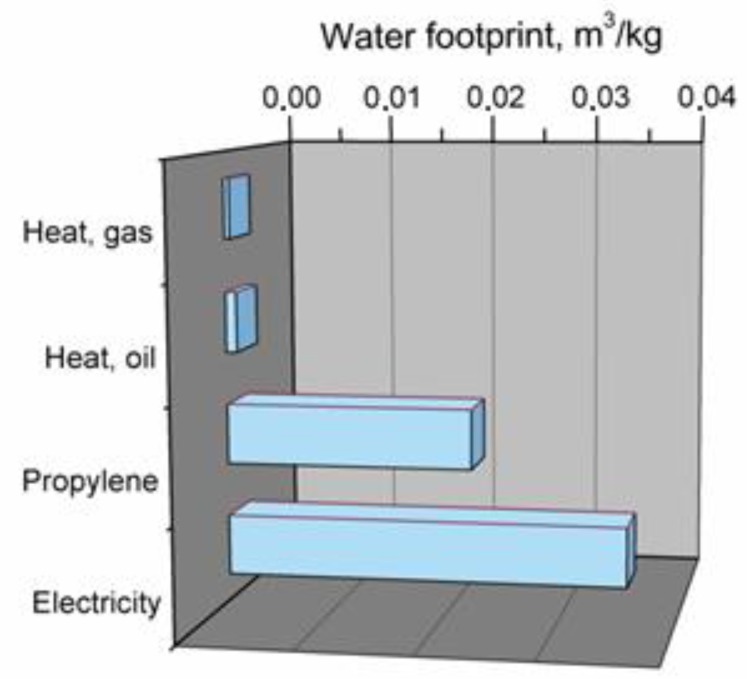
Main components of production system generating water footprint of polypropylene.

The conducted analysis enabled the determination of crucial components of the PP production process, which most significantly affects its water footprint. Results are presented in Figure 6.

As can be seen from the presented chart, the water footprint of PP is mainly affected by the use of electricity and production of propylene from crude oil, which is a main component used during manufacturing of PP in the polymerization process. These elements constitute 97% of total PP water footprint in analyzed production system. Other elements, which are related to generation of heat required for production, have a noticeably lower load for water usage.

#### 3.2.2. Poly(lactic acid)

**Figure 7:**
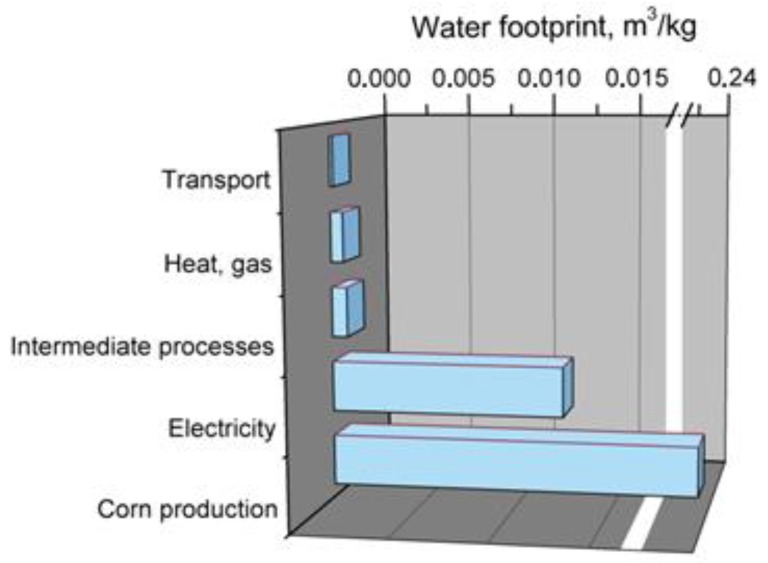
Main components of production system generating water footprint of poly(lactic acid).

Figure 7 presents the main elements affecting total water footprint of PLA production.

Noticeably, the biggest burden affecting the water footprint of PLA is production of maize used as a main raw material in manufacturing of PLA. It stands for ~94% of total water footprint of PLA. Such a phenomenon is associated with the fact that water footprint for crops and food products is noticeably higher than for various petroleum-based chemicals. Use of electricity stands for ~5% of PLA water footprint and is around two times lower than for PP. Other elements, such as transport, heat generation, and intermediate chemical processes are responsible for only 1% of total water footprint of this bioplastic.

#### 3.2.3. Cotton Fibers

**Figure 8:**
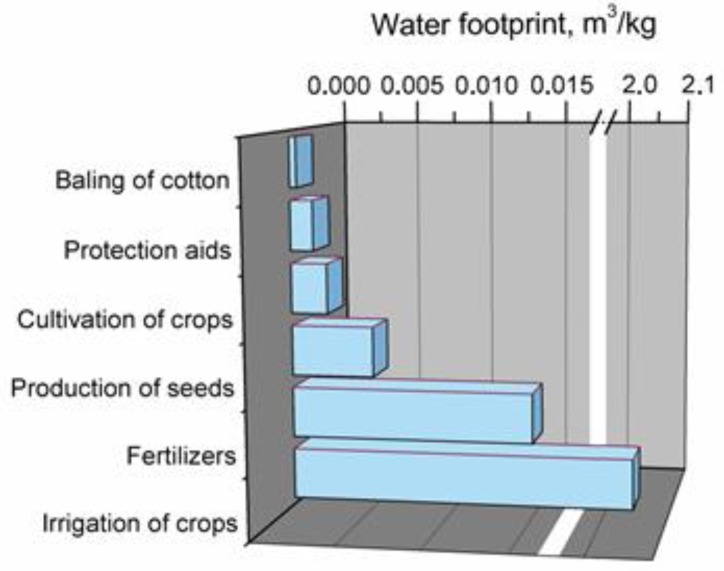
Main components of production system generating water footprint of cotton fibers.

The main components of cotton fibers’ production process and their effects on water footprint, determined during performed analysis, are presented in Figure 8.

In the case of cotton fibers, basically the whole water footprint is related to the raw materials and their processing. The most significant is irrigation of cotton crops, which, with a value of 2.05 m^3^/kg, accounts for almost 99% of total water footprint. The production of seeds, cultivation of cotton, protection of crops (herbicides, pesticides, etc.), packing of cotton, and most of all application of fertilizers are, among other factors, major influences on this parameter.

#### 3.2.4. Jute Fibers

**Figure 9:**
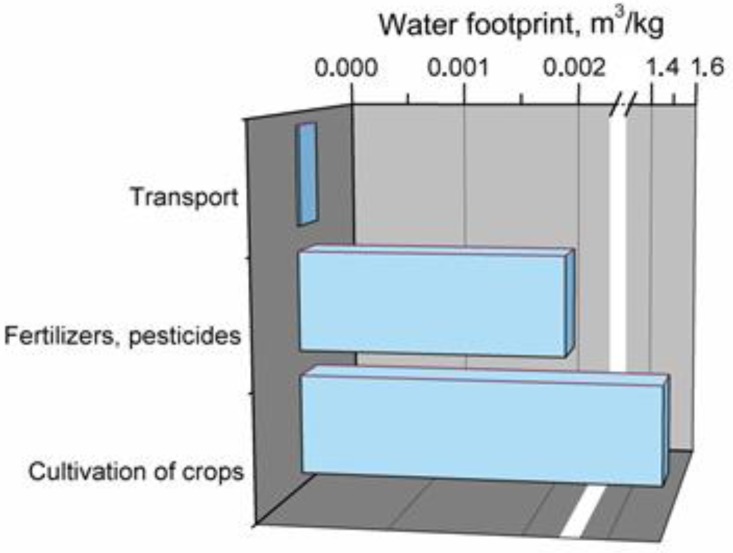
Main components of production system generating water footprint of jute fibers.

Performed analysis enabled determination of water footprint of the jute fibers production process. Results are presented in Figure 9.

It was determined that, just as in the case of cotton fibers, the most significant impact is observed for raw materials, cultivation of crops required for manufacturing of jute fibers and their processing, which accounts for almost 99% of total water footprint. Among other factors were the distinguished application of fertilizers and pesticides and transport influencing this parameter.

#### 3.2.5. Kenaf Fibers

**Figure 10:**
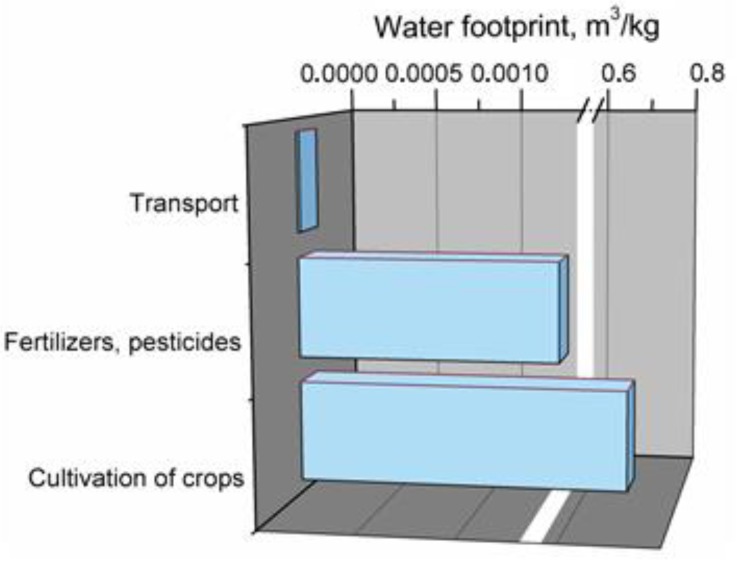
Main components of production system generating water footprint of kenaf fibers.

Figure 10 shows the main elements affecting total water footprint of kenaf fibers production.

The distribution of shares in total water footprint for kenaf fibers production is very similar to that of jute fibers. The main component is cultivation of crops, whose high share is mainly affected by need for irrigation. Application of fertilizers and pesticides as well as transport account for only ~1% of total water footprint.

#### 3.2.6. Glass Fibers

**Figure 11:**
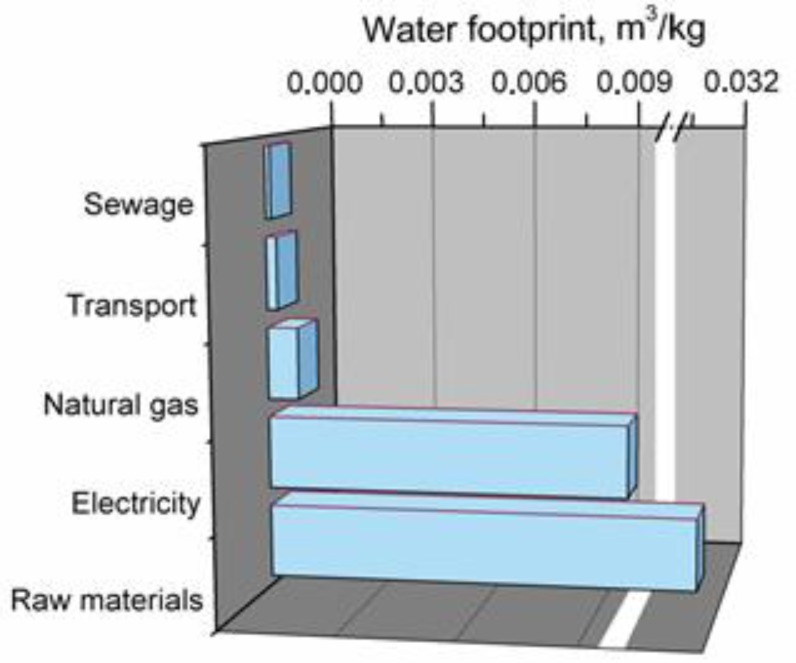
Main components of production system generating water footprint of glass fibers.

Figure 11 shows the main elements affecting total water footprint of glass fibers production.

In the case of glass fibers, the most significant impact on water footprint (~74%) is observed for applied raw materials, e.g., silica, aluminum oxide, boric acid, clays, fluorite, lime, and other additives and modifiers. Another factor with significant share in total water footprint is the use of electricity, accounting for ~23% of this indicator. Just as in the cases of the above mentioned plastics, share of other elements, in this case natural gas, transport, and sewage, is noticeably lower.

#### 3.2.7. Summary

**Figure 12:**
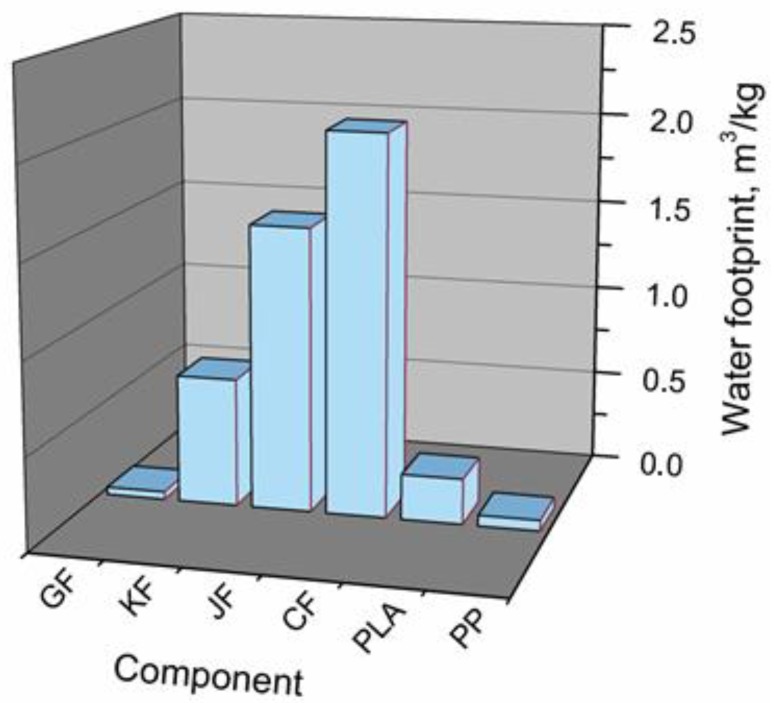
Comparison of water footprint values for all applied raw materials.

Performed analysis resulted in the determination of water footprints for all analyzed raw materials, which are presented in the Figure 12. It can be seen that the highest values of this indicator were observed for natural fibers, 0.70, 1.55, and 2.07 m^3^/kg for kenaf fibers, jute fibers, and cotton fibers, respectively. Such a phenomenon is associated with the large quantity of water required for the cultivation of crops used in production of fibers. For glass fibers, the water footprint was significantly lower—0.041 m^3^/kg; therefore, considering only water footprint, glass fibers can be considered a lower environmental burden than natural fibers. Nevertheless, for comprehensive analysis many other factors have to be considered.

Among analyzed polymeric materials, a higher water footprint was determined for PLA (0.248 m^3^/kg), which is associated with maize cultivation. For PP, the value of water footprint was ~4 times lower and equaled 0.059 m^3^/kg.

It is also important to mention that in the presented work we did not investigate the influence of the varying materials’ properties, such as degree of crystallinity, glass transition temperature, or melting point, on the water footprint of resulting products. We are fully aware that these parameters affect the amount of energy required during processing of plastics; however, considering water footprint, they are not so important, which was confirmed by Larson et al. [60] in the report on water footprint assessments of dehydrated onion products microirrigation systems. Authors analyzed the application of PVC, PP, and linear low-density polyethylene (LLDPE) in manufacturing of pipes, tubes, and other molded components. Total water footprints of these elements were in the range 0.011–0.019 m^3^/kg. Authors also calculated the operational water footprint values of analyzed materials, related to the extrusion and injection molding, whose values could be affected by changing crystallinity or melting point of raw materials. Values of operational water footprint were from 0.5 to 4.1 liters/kg, from which 14% to even 65% were related to the domestic water use or overhead, which includes water consumed or polluted related to toilets, cleaning, kitchens, gardening, and laundry. Therefore, performed analysis was very detailed and included even the slightest uses of water. Considering that in our case water footprint values of PP and PLA were determined as 59 and 248 liters/kg, we believe that even significant changes in crystallinity could be considered insignificant for the total water footprint of final pallet.

## 4. Conclusions

The results of the presented study show that, in terms of water footprint, biocomposites, considered widely as very environmentally-friendly materials, are a significantly greater burden to the environment than conventional plastics and composites filled with glass fiber. Such results may be surprising, but it is very important to remember that they are referring only to one particular aspect of these materials—use of water. Therefore, they should not be used as an individual indicator of materials’ usefulness and friendliness, but only as an input element for analysis including a wider spectrum of environmental factors. Nevertheless, we believe that there are multiple ways to reduce the water footprint of plastics, among them should be mentioned:

- proper management of waste generated during plastics production, which could be recycled and partially introduced into stream of raw materials, reducing the use of primary resources,
- introduction of continuous processes instead of periodic ones, which could enhance the ecological and economical aspects of production processes, e.g., reduce the amount of water required for production and additional operations, such as purification of fillers modified in a periodic manner with the use of various organic or inorganic solvents,
- creating added value for waste materials, which are currently not utilized, e.g., various types of waste from food industry, which could be applied as source of lignocellulosic fillers in the manufacturing of wood polymer composites.

To sum up, we believe that the determination of water footprint as an indicator of environmental impact may be a very useful tool for the plastics industry, because it enables more comprehensive analysis of various materials and production processes from a different point of view. Such analysis could be definitely very helpful for the engineering of processes and materials with the lowest possible impact on the natural environment.

## Figures and Tables

**Table 1:** Composition variants of analyzed EUR-pallet.

Component	Variant					
	PP	PP/PLA	PP/CF	PP/JF	PP/KF	PP/GF
	Content, wt %					
Polypropylene	100	70	70	70	70	90
Poly(lactic acid)	-	30	-	-	-	-
Cotton fibers	-	-	30	-	-	-
Jute fibers	-	-	-	30	-	-
Kenaf fibers	-	-	-	-	30	-
Glass fibers	-	-	-	-	-	10
